# miRNAs: EBV Mechanism for Escaping Host’s Immune Response and Supporting Tumorigenesis

**DOI:** 10.3390/pathogens9050353

**Published:** 2020-05-08

**Authors:** Snježana Židovec Lepej, Maja Matulić, Paula Gršković, Mirjana Pavlica, Leona Radmanić, Petra Korać

**Affiliations:** 1 Department of Immunological and Molecular Diagnostics, University Hospital for Infectious Diseases “Dr. Fran Mihaljević”, 10000 Zagreb, Croatia; szidovec@gmail.com (S.Ž.L.); leona.radmanic@gmail.com (L.R.); 2 Division of Molecular Biology, Department of Biology, Faculty of Science, University of Zagreb, 10000 Zagreb, Croatia; maja.matulic@biol.pmf.hr (M.M.); paula.grskovic@biol.pmf.hr (P.G.); mpavlica@zg.biol.pmf.hr (M.P.)

**Keywords:** miRNA, EBV, carcinoma, lymphoma

## Abstract

Epstein-Barr virus (EBV) or human herpesvirus 4 (HHV-4) is a ubiquitous human oncogenic virus, and the first human virus found to express microRNAs (miRNAs). Its genome contains two regions encoding more than 40 miRNAs that regulate expression of both viral and human genes. There are numerous evidences that EBV miRNAs impact immune response, affect antigen presentation and recognition, change T- and B-cell communication, drive antibody production during infection, and have a role in cell apoptosis. Moreover, the ability of EBV to induce B-cell transformation and take part in mechanisms of oncogenesis in humans is well known. Although EBV infection is associated with development of various diseases, the role of its miRNAs is still not understood. There is abundant data describing EBV miRNAs in nasopharyngeal carcinoma and several studies that have tried to evaluate their role in gastric carcinoma and lymphoma. This review aims to summarize so far known data about the role of EBV miRNAs in altered regulation of gene expression in human cells in EBV-associated diseases.

## 1. Introduction

Epstein-Barr virus (EBV) or human herpesvirus 4 (HHV-4) is a ubiquitous human oncogenic virus that belongs to the family *Herpesviridae*, subfamily *Gammaherpesvirinae*, and genus *Lymphocryptovirus* [1]. Since its first description in 1964 and subsequent recognition of its ability to induce B-cell transformation in vitro, EBV has been extensively used as a model for research focusing on fundamental mechanisms of oncogenesis in humans. It is also used as a model in devising diagnostic, therapeutic, and prevention strategies in malignant diseases [2,3,4,5]. Approximately 95% of the human population is infected with EBV and will remain carriers of the virus for the rest of their lives. By modulating its own transcriptional patterns during lytic and latent stages of infection, EBV establishes a lifelong persistence in both immunocompetent and immunocompromised hosts and modifies the host’s immune system effector mechanisms [6]. However, in the context of immunosuppression, regardless of the specific cause, the immune system fails to efficiently control EBV replication [7]. Subsequently, latent infection with EBV may be associated with development of various malignancies originating from epithelial cells, lymphocytes, and mesenchymal cells, including posttransplant B-cell lymphomas, Hodgkin’s and non-Hodgkin’s lymphomas, diffuse large B-cell lymphoma, Burkitt’s lymphoma, natural killer (NK)/T-cell lymphoma, nasopharyngeal carcinoma (NPC), and gastric carcinoma (GC) [7,8].

MicroRNAs (miRNA) are small, non-coding RNA molecules of cellular or viral origin that consist of 18–22 nucleotides and play an important role in regulation of gene expression. Consequently, they affect key events in cell biology such as proliferation, apoptosis, and lipid metabolism. By binding to messenger RNAs (mRNAs), miRNAs induce degradation of mRNAs or inhibition of translation, thus reducing levels of expression of target genes. Virus-encoded miRNAs (v-miRNAs) are considered an important non-immunogenic tool for post-transcriptional regulation of both host and viral gene expression in infected cells [9]. The majority of literature data on v-miRNAs, particularly in the context of tumorigenesis, focuses on v-miRNAs in EBV, Kaposi’s sarcoma-associated herpesvirus (KSHV/HHV8), human papillomaviruses (HPV), hepatitis C virus (HCV), hepatitis B virus (HBV), and Merkel Cell Polyomavirus (MCPyV) [9,10,11]. EBV was the first virus shown to express miRNAs [12]. EBV-encoded miRNAs (EBV miRNAs) target both viral and cellular mRNAs in infected cells, extending their role beyond regulating various stages of the EBV replication cycle. They influence cellular proliferation and apoptosis and play a part in driving diverse molecular pathways of oncogenesis and evading innate and adaptive immune responses. The aim of this review is to summarize current views on the role of EBV miRNAs in altered gene expression associated with immune evasion and tumorigenesis.

## 2. EBV miRNAs

**Figure 1:**
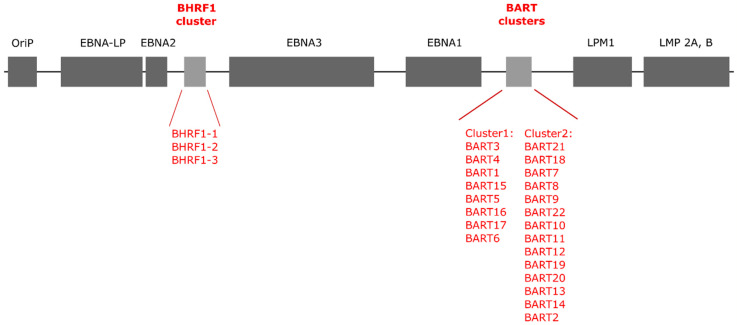
Schematic presentation of the Epstein–Barr virus genome structure (based on References [12,20,21]).

EBV genome is a double-stranded DNA molecule that consists of 175 kbp containing nearly 100 genes and coding for 44 microRNAs [13,14,15]. It was first sequenced in 1984 by using M13 libraries made from viral *Eco*R I and *Bam*H I fragments gathered after sonification [16]. Today, after more than 100 EBV genomes from tumor cells samples and healthy individuals’ tissue have been sequenced, the complete EBV genome can be found in the NCBI GeneBank [17]. In 2004, Pfeffer et al. first described two clusters in the EBV genome responsible for production of EBV miRNAs [12]. The first cluster was found in the sequence for *Bam*H I fragment H rightward open reading frame 1 (BHRF1) mRNA. It produces three miRNA precursors (ebv-miR-BHRF1-1, -2, and -3) and, subsequently, four mature miRNAs [18]. The second cluster is BamH I-A region rightward transcript (BART) and it encodes 22 miRNA precursors (ebv-miR-BART1-22) of 40 mature miRNA molecules [12,19] (Figure 1).

Expression of BHRF1 miRNAs is latency stage-dependent (they are mainly expressed in type III latency), while BART miRNAs are transcribed in all latency stages [12]. Despite the coordinated expression of each miRNA cluster, significant differences in the expression levels of individual EBV miRNAs in the same type of human cells (as high as 50-fold) have been observed [12,22]. It has been suggested that different genotypes as well as genomic variants of EBV could be associated with different patterns of individual miRNA expression in infected cells [23,24,25]. A number of studies on EBV miRNA biosynthesis (reviewed by Wang et al., 2018) suggest that EBV gene products are not necessary for v-miRNA production. This proposition indicates that the molecular mechanisms responsible for the synthesis of v-miRNAs and cellular miRNAs in host cells are similar [20]. Analysis of EBV genomic sequences in gastric carcinoma and EBV-associated lymphoma has showed that despite the genetic diversity in almost the entire EBV genome, the regions encoding miRNAs are highly conserved [26,27].

## 3. The Role of EBV miRNAs in Immune Evasion

Alongside immune evasion strategies mediated by host miRNAs and viral glycoproteins, EBV miRNAs are another means by which the virus successfully avoids effector mechanisms of the host’s immune system [28]. The main strategies for immune evasion used by EBV miRNAs are the following:

### 3.1. Pattern-Recognition Receptor-Mediated Signaling Pathways and Interferons

**Figure 2:**
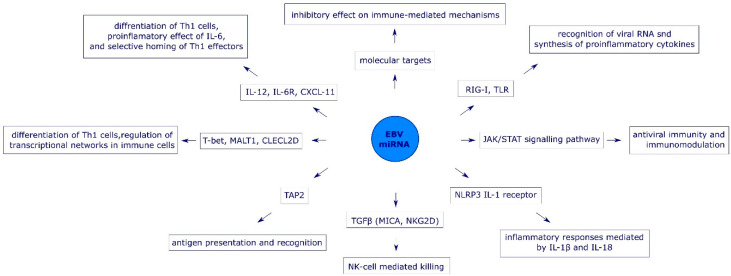
The role of EBV miRNAs in the immune evasion.

EBV interferes with efficient initiation of innate immune response at the very first step, e.g., by targeting expression of pattern recognition receptors (PRR). The impaired expression of PRR affects subsequent signal transduction as well as cytokine synthesis [29]. The two main targets for EBV miRNAs are retinoic acid-inducible protein 1 (RIG-I) receptors (mediated by miR-BART6-3p) and Toll-like receptors [20,30]. The lack of EBV molecular pattern recognition by PRR is associated with the absence of the JAK (Janus kinase)-STAT (Signal Transducer and Activator of Transcription)-mediated transduction pathway. The ineffectiveness of this signaling pathway leads to the impaired synthesis of type I interferons (IFNs) and other pro-inflammatory cytokines that are essential for innate immune responses [31]. Studies on nasal NK-cell lymphoma (NNL) cells showed that EBV miRNAs (miR-BART20-5p and miR-BART8) impact the signal transduction pathway for IFN-γ by targeting STAT1, which enables the virus to avoid antiviral activity of both type I and type II IFNs [32] (Figure 2).

### 3.2. Natural Killer (NK) Cells

The role of EBV miRNAs in the evasion of NK-cell-mediated responses has been analyzed in nasopharyngeal carcinoma cells in vitro. Interaction between the natural killer group 2 member D (NKG2D) receptor on NK cells and major histocompatibility complex class I chain-related peptide A (MICA) is considered the key step in the recognition and killing of cancer cells. Expression of MICA in NPC is positively regulated by transforming growth factor β-1 (TGF-β1). Wong et al. (2018) showed that miR-BART7 reduces the expression of TGF-β1 in NPC cells and impairs the NK-cell-mediated recognition of virus-infected cells [33] (Figure 2).

### 3.3. Inflammasome

Immune evasion strategies employed by EBV also target NLR family pyrin domain-containing 3 (NLRP3) inflammasomes, which are responsible for inflammatory responses mediated by IL-1β and IL-18 upon recognition of viral antigens. The activity of inflammasomes is targeted directly at the level of NLRP3 expression (via miR-BART15), or indirectly, by targeting the IL-1 receptor itself (via miR-BHRF1-2-5p) [34,35] (Figure 2).

### 3.4. Cytokines and Chemokines

miR-BART1, miR-BART2, miR-BART10, and miR-BART22 suppress efficient CD8+ T-cell-mediated antiviral immune response by targeting IL-12, a pro-inflammatory cytokine responsible for differentiation of naive type 1 helper T-cell (Th1 cells) to mature Th1 cells, increased synthesis of IFN-γ, activation of NK- and T-cells as well as inhibition of angiogenesis [36,37]. In addition, miR-BART6-3p interferes with biological activity of IL-6 by targeting expression of the IL-6 receptor [38]. EBV miRNAs also enable the virus to evade Th1-mediated antiviral immunity by modulating expression of chemokines. For example, miR-BHRF1-3 targets an IFN-inducible chemokine CXCL11 (CXC-chemokine ligand 11) responsible for the selective homing of Th1 effector cells and NK-cells to the sites of infection [20,39] (Figure 2).

### 3.5. Antigen Presentation

EBV miRNAs impair mechanisms of specific immunity by affecting adequate antigen recognition at the level of antigen processing (reduced expression of lysosomal enzymes), transport of processed antigenic peptides to major histocompatibility complex (MHC) molecules (by targeting peptide transporter subunit TAP2), and antigen presentation (reduction of lymphocyte antigen 75 expression on dendritic cells) [20,37,40,41,42] (Figure 2).

### 3.6. Specific Cellular Immunity

T-cell-mediated immunity can maintain long term immune control over EBV replication (for >50 years in some individuals) while clinical consequences associated with EBV infection in persons with impairment of T-cell development or function are shown to be very severe. This suggests that virus-specific T-cell responses represent the main means of protective immunity in EBV infection [37]. EBV miRNAs specifically target host genes coding for key regulators of T-cell responses including T-bet (miR-BART20-5p), Mucosa-associated lymphoid tissue lymphoma transport protein 1 (MALT1) (miR-BHRF1-2-5p), and C type lectin superfamily 2 member D (CLEC2D) (miR-BART1-3p and miR-BART3-3p) [43] (Figure 2).

T-bet belongs to the T-box family of transcription factors that are the main enhancers of the Th1 differentiation pathway and subsequent Th1 cell-specific IFN-γ synthesis, which are important for efficient antiviral immunity. Inhibition of T-bet translation (with subsequent suppression of p53) by EBV miRNAs, originally shown in invasive nasal NK/T-cell lymphoma cells, is supposedly associated with the inhibition of Th1 differentiation pathways. As a result, the control of EBV replication is less efficient [43].

However, T-bet also regulates transcriptional networks that are common among other types of immune cells (including dendritic cells and innate lymphoid cells) and is currently considered to have an important role in bridging innate and adaptive immunity (for review see Lazarevic et al., 2017) [44].

In addition, T-bet acts as a selective repressor of transcriptional pathways associated with type I IFNs subsequent to IFN-γ-induced signaling [44,45]. Therefore, EBV miRNAs-mediated inhibition of T-bet’s biological activity may have a significantly broader effect on the evasion of antiviral immunity in EBV infection than originally thought.

## 4. The Role of EBV miRNAs in Tumorigenesis

EBV association with development and progression of malignant tumors is well known, especially its frequent infection of lymphoma and carcinoma cells. Nevertheless, the role of EBV miRNAs in tumorigenesis has only recently come into focus.

### 4.1. EBV miRNAs in Lymphoma

EBV miRNAs have been linked to development and progression of various types of lymphoma, such as acquired immunodeficiency syndrome-related diffuse large B-cell lymphoma (AIDS-related DLBCL), EBV-positive DLBCL, Burkitt lymphoma (BL), nasal NK/T-cell lymphoma (NNL), Hodgkin’s lymphoma (HL), posttransplant lymphoproliferative disorder (PTLD), and others [23].

miR-BHRF1-3 was found to be highly expressed in AIDS-related DLBCL compared to EBV-positive BL and EBV-negative DLBCL [39]. miR-BHRF-1-1 was detected in all samples of EBV-associated primary central nervous system PTLD (pCNS PTLD) and mir-BHRF-1-2 was found in about 50% of the same pCNS PTLD patients [46].

EBV-miRNAs belonging to BART clusters were found in pCNS PTLD, but with varying expression of different BART miRNAs [46]. Furthermore, miR-BART7, miR-BART22, miR-BART10, miR-BART11-5p, and miR-BART16 were found to be most prominently expressed in EBV-positive DLBCL not associated with AIDS [47]. Only EBV miRNAs from BART clusters were found in endemic BL (eBL) [48]. miR-BART7, miR-BART5, miR-BART11-5p, miR-BART1-5p, and miR-BART19-3p were found in NNL, as well as miR-BART21 and miR-BART22, previously found in EBV-positive carcinomas [49,50]. Expression of BART miRNAs have also been observed in HL [12].

The presence of diverse EBV miRNAs in different types of lymphoma was analyzed not only in patient samples, but it was also researched through cell line-based studies. In BL41/95 cell line, derived from BL, BHRF miRNAs were detected [12]. The study by Ambrosio et al. in which BL-derived cell line Akata was used as a model, revealed that the expression of PTEN and IL6 receptor subunits was lower in the presence of miR-BART6-3p and was restored if the cells were simultaneously transfected with miR-BART6-3p inhibitor [48]. Zhou et al. found that Ramos cell line (derived from EBV-negative BL) transfected simultaneously with oligonucleotides mimicking cellular miRNA-142 and miBART6-3p, displayed lower expression of PTEN compared to negative control and cells transfected with the same oligonucleotides separately. This suggests that miR-BART6-3p downregulates PTEN, a tumor suppressor that regulates the PI3K/Akt pathway, in cooperation with cellular miRNA-142 [51]. Moreover, in two cell lines derived from lymphomas of NK cell origin (YT and NK92), it was shown that miR-BART20-5p was responsible for downregulation of T-bet, and therefore p53, and IFN-γ [43].

### 4.2. EBV miRNAs in Carcinoma

EBV is also known to be associated with the development of NPC and GC. Recently, the role of EBV miRNAs was thoroughly studied in these malignancies. BART miRNAs were first found in NPC, xenografted and propagated in nude mice [52,53], and subsequently in NPC patient biopsies [54]. They were found to be highly expressed in NPC and GC, but BHRF1 miRNAs were generally not present in those entities. At least 105 host genes were shown to be regulated by BART miRNAs during carcinoma development [55], but genes recognized as EBV miRNAs’ targets in B-cell lymphomas were not confirmed in carcinomas. Increased expression of BART miRNA clusters and individual BART miRNAs correlated with higher tumor grade and poor patient survival in NPC and GC.

Generally, it is believed that BART miRNAs in carcinoma act in synergy or obtain significant effects by combining their individual activities, in cooperation or competition with cellular miRNAs. It was shown that BART miRNAs target numerous transcripts of different genes, thus deregulating various downstream molecules and signaling pathways [56]. Consequently, BART miRNAs allow infected cells to avoid apoptosis by inactivating different pro-apoptotic molecules, influence cell proliferation, inhibit the expression of regulatory tumor suppressors, mediate cancer metabolism, stimulate cell migration and metastasis, inhibit cell differentiation, and manage immune evasion and regulation of the virus latency through coordination of cellular and viral signaling pathways [49,57,58] (Table 1).

BART miRNAs also modulate host cell pathways by mimicking cellular miRNAs. Although this feature is still not explored enough, several BART miRNAs were shown to have “seed” sequences similar to those of cellular miRNAs: miR-BART5 compared to miR-18a and miR-18b [66,79], miR-BART9 to miR-200a and miR-141 [72], miR-BART15-3p to miR-223-3p, and miR-BART18-5p to miR-26a [35,80].

Overall, BART miRNAs are highly expressed in NPC and GC types associated with EBV infection where they act synergistically and have redundant activities, but also possibly differ in their target genes in different intracellular milieus.

## 5. Conclusions

We envision that further detection of cellular processes affected and regulated by EBV miRNAs will contribute to better understanding of the role of viral non-coding RNAs in the development of virus-induced cancers in humans. In case of EBV, despite extensive research, there are currently no antiviral drugs or EBV-vaccines approved for use in humans. In years to come, better evaluation and understanding of the viral miRNA mechanisms might reveal new biomarkers and potential therapeutic targets.

## Figures and Tables

**Table 1:** Targets of BART miRNAs and processes affected by their interactions in carcinoma cells.

BART	BART Target	Affected Process	Study	Method
1	PTEN	migration, signaling	Cai et al. 2015a, Cai et al. 2015b [59,60]	reporter assay
1-5p	α1subunit AMPK	metabolism	Lyu et al. 2018 [61]	reporter assay, biopsies, cell culture
1-5p	LMP1	viral latency	Lo et al. 2007 [62]	reporter assay
3	DICE1	apoptosis	Kang et al. 2015, Lei et al. 2013 [63,64]	reporter assay, PAR-CLIP
3	CASZ1a	apoptosis	Kang et al. 2015 [63]	PAR-CLIP, reporter assay
4-5p	BID	apoptosis	Shinozaki-Ushiku et al. 2015 [65]	reporter assay, biopsies
5	PUMA	apoptosis	Choy et al. 2008 [66]	reporter assay
5-3p	p53	apoptosis	Zheng et al. 2018 [67]	reporter assay
5-5p	ATM	DNA repair	Lung et al. 2018 [68]	reporter assay
6-3p	LOC353103 RNA	anti-migration	He et al. 2016 [69]	cell culture, reporter assay
6	OCT1	apoptosis	Kang et al. 2015 [63]	PAR-CLIP, reporter assay
6	Dicer	apoptosis	Kang et al. 2015, Iizasa et al. 2010 [63,70]	PAR-CLIP, reporter assay
7-3p	PTEN	migration, signaling	Cai et al. 2015a, Cai et al. 2015b [59,60]	reporter assay
7-3p	ATM	DNA repair	Lung et al. 2018 [68]	reporter assay
8-3p	RNF38	signaling, migration	Lin et al. 2018 [71]	biopsy sequencing, reporter assay
8	ARID2	unknown	Kang et al. 2015 [63]	PAR-CLIP, reporter assay
9	E CAD	migration	Tsai et al. 2017, Hsu et al. 2014 [24,72]	reporter assay, biopsies
9-3p	ATM	DNA repair	Lung et al. 2018 [68]	reporter assay
10	BTRC	signaling, migration	Zeng et al. 2014, Yan et al. 2015 [57,73]	reporter assay, biopsies
10-3p	DKK1	signaling, migration	Min et al. 2019 [74]	reporter assay
11	trFOXP1	immune evasion, differentiation	Song et al. 2016 [75]	reporter assay, biopsies
13	NKIRAS2	signaling	Xu et al. 2019 [76]	biopsies, cell culture
14-3p	ATM	DNA repair	Lung et al. 2018 [68]	reporter assay
15-3p	BRUCE	anti-apoptosis	Choy et al. 2013 [77]	reporter assay, WB
16	CRBBP	apoptosis	Kang et al. 2015 [63]	PAR-CLIP, reporter assay
16	SH2B3	apoptosis	Kang et al. 2015 [63]	PAR-CLIP, reporter assay
16	TOMM22	apoptosis	Kang et al. 2015 [63]	PAR-CLIP, reporter assay
16	LMP1	viral latency	Lo et al. 2007 [62]	reporter assay
17-5p	LMP1	viral latency	Lo et al. 2007 [62]	reporter assay
22	PAK2	apoptosis	Kang et al. 2015 [63]	PAR-CLIP, reporter assay
22	TP53INP1	apoptosis	Kang et al. 2015 [63]	PAR-CLIP, reporter assay
22 and cluster II	NDRG1	metastasis, differentiation	Kanda et al. 2015 [78]	microarray reporter assay
